# Female Reproductive Tract Organoids: Applications from Physiology to Pathology

**DOI:** 10.3390/biom15070925

**Published:** 2025-06-24

**Authors:** Xinyu Wang, Diqi Yang, Hui Peng

**Affiliations:** School of Tropical Agriculture and Forestry, Hainan University, Haikou 570228, China; 20213007854@hainanu.edu.cn

**Keywords:** female reproductive tract, organoid, hydrogel, reproductive physiology, reproductive disorder

## Abstract

The female reproductive tract (FRT) serves as the core of human reproduction, and its health is directly related to population quantity and family happiness. The high incidence rate of female reproductive tract diseases globally poses a severe threat to women’s health. Nevertheless, the exploration of its physiological functions and pathological mechanisms still lacks satisfactory research models. Organoids, as an emerging technology, not only circumvent numerous ethical issues existing in in vivo experiments but also precisely replicate the morphological structure and characteristics of the simulated tissues. The purpose of this article is to summarize the basic paradigms of organoid establishment and their applications in female reproductive research. Specifically, this article summarizes the cell sources, extracellular scaffolds, and culture media used in the establishment of organoids. It also describes the applications and future development prospects of female reproductive tract organoids established in current research in physiological and pathological studies. The importance of organoid technology in the female reproductive tract research cannot be ignored. It has opened up new avenues for research in this field and greatly promoted the exploration of female reproductive health and disease mechanisms.

## 1. Introduction

The female reproductive tract (FRT) is exquisitely embedded in the pelvic region of the human body, extending from approximately the exterior to the deep interior, forming a complex and elaborate structural network [1]. The ovary, the fallopian tube, the uterus, and the cervix, as the four most critical components of the reproductive tract, assume the principal functions of the reproductive tract [2]. The ovary, serving as the principal gonad of females, is accountable for generating oocytes (ova) and secreting hormones. In each menstrual cycle, the mature oocyte released from the ovary is captured by the fimbriae of the fallopian tube and conveyed to the interior of the tube. Within the ampulla of the fallopian tube, sperm encounters the oocyte, and the fertilization process is accomplished. After the fertilized egg is transported to the uterine cavity by the fallopian tube, the endometrium of the uterus undergoes a succession of intricate physiological alterations to furnish appropriate conditions for the implantation of the fertilized egg. The fertilized egg embeds and takes hold in the endometrium, giving rise to the placenta. Prior to the implantation of the fertilized egg, the cervix remains closed, thereby guaranteeing the stability and security of the intrauterine environment. Nevertheless, during the period of childbirth, the cervix gradually softens and commences dilation, opening up a passageway for the expulsion of the fetus. The ovary, the fallopian tube, the uterus, and the cervix each undertake distinctive physiological functions and successively play pivotal roles in the female reproductive process [3].

The normal functioning of the female reproductive tract is closely associated with the neuro–humoral–immune regulatory network. Under the action of multiple systems, the reproductive tract is situated in an internal environment involving the dynamic equilibrium of various substances [4,5,6]. Once such a balance is disrupted by internal or external factors, the reproductive tract will unavoidably exhibit a pathological state [7]. For example, the expression and levels of brain-derived neurotrophic factor (BDNF) are intimately associated with ovarian development, follicular growth, oocyte maturation, endometrial cell proliferation, and neural development. Meanwhile, the dysregulation of BDNF expression and circulating concentrations may give rise to premature ovarian failure, endometriosis, and several reproductive cancer disorders [8]. Insulin-like growth factor binding protein-1 (IGFBP-1) plays pathophysiological roles in preeclampsia, polycystic ovary syndrome, and trophoblastic and endometrial tumors [9]. The dysregulation of the function of FRT macrophages and estrogen responsiveness might be implicated in the genesis of ovarian cancer and endometriosis [10]. Furthermore, alterations in the external environment, primarily denoting changes in physicochemical parameters, such as the oxygen partial pressure (pO2), pH value, and temperature within the female reproductive tract, frequently exert detrimental influences on sperm motility during fertilization and embryo development, thereby often resulting in pregnancy failure [11].

As the incidence of a series of reproductive tract disorders has been escalating year after year, it has become increasingly imperative to comprehend how the organism governs the functions of the female reproductive tract (FRT) and the associated cellular and molecular mechanisms. Constrained by the complexity of the FRT and ethical concerns, research on reproductive disorders remains rather sluggish [12]. Under such circumstances, a growing number of researchers have focused their sights on alternatives. Can an in vitro model that can perfectly simulate the reproductive tract be constructed?

**Figure 1:**
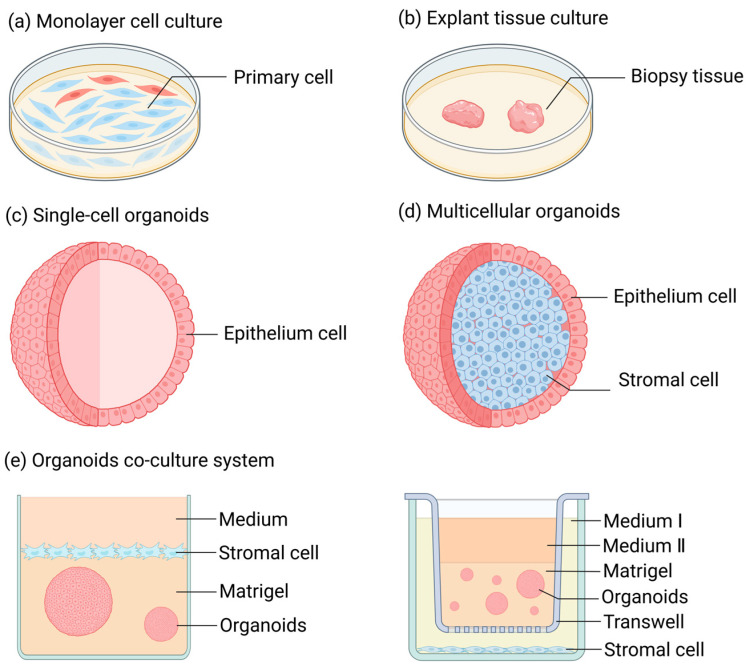
Research methods for female reproductive tract diseases. (**a**) Monolayer cell culture: A method of cell culture in animal cells where cells rapidly spread and start mitosis once adhered to the surface, gradually forming a dense monolayer of cells. (**b**) Tissue block culture: A method of culturing where freshly isolated, highly viable tissue is cut into small pieces and inoculated into culture flasks for growth. (**c**) Single-cell organoids: They are derived from a single type of stem cell or purified primary cells. Prior to organoid culture, these initial cells exist in either an undifferentiated state (stem cells) or a highly differentiated state (mature somatic cells, such as endometrial epithelial cells), featuring a singular and stable transcriptional level. (**d**) Multicellular organoids: Cell aggregates consisting of multiple cell types. These refer to multiple types of cells or a variety of highly differentiated primary somatic cells that are obtained by inducing the differentiation of stem cells through in vitro culture techniques prior to the initiation of organoid culture. (**e**) Organoid co-culture system: A system where different types of organoids and cells are co-cultured to simulate the interactions between different tissues or organs in vivo.

Over an extended period of time, when conducting research on FRT diseases, one typically had to make a choice between monolayer cell culture (Figure 1a) and tissue culture (Figure 1b) [13,14,15,16]. Monolayer cell culture is a conventional two-dimensional adherent cell culture approach, possessing advantages like simplicity of the method and low cost. The majority of cells cultivated through this method are primary cells derived from normal tissue biopsies [17] or cells that have undergone carcinogenesis (or have been immortalized) [18]. The former is prone to losing the original polarity and characteristic features of the cells during the culturing process, such as hormone responsiveness, and it is unable to proliferate infinitely. However, the latter, although having the advantage of unlimited proliferation, cannot represent cells in a normal physiological state due to the differences in gene expression of its cells compared to normal circumstances. Tissue culture is a three-dimensional and stereoscopic culture mode characterized by diverse cell populations, intercellular interactions, and tissue architectures [19,20]. However, the time of tissue culture is typically very brief, rendering it challenging to support research over an extended period.

The three-dimensional organoids developed by Sato and Clevers (Figure 1c,d) have offered novel insights for the investigation of the physiological and pathological mechanisms of FRT [21]. Organoids are three-dimensional cell structures with self-organizing ability that can manifest certain specific functions of an organ in the body [22]. The cell sources constituting organoids encompass pluripotent stem cells (PSCs), primary cells, and cell lines [23]. Under specific culturing conditions, the organoids formed by PSCs can replicate the “organogenesis” process, such as that of the intestines, liver, lungs, and other organs [24,25,26]. Meanwhile, organoids composed of primary cells derived from tissues can be subjected to long-term culture and expansion under optimal culturing conditions, successfully simulating the cellular physiological state of organs in vivo [23].

Organoids and their co-culture models (Figure 1e) have emerged as crucial tools for researching reproductive physiology and pathology over the past decade, significantly promoting our comprehension of the structure and function of the reproductive tract [27,28,29,30]. In contrast to traditional two-dimensional (2D) culture techniques, organoids mark revolutionary progress. Organoids are capable of recapitulating three-dimensional (3D) tissue architectures, cellular heterogeneity, and physiological functions [31,32]. Differing from 2D monolayers, organoid models can self-assemble into microenvironments with spatial organization, thus preserving the dynamics of stem cells and patient-specific phenotypes [33]. They enable the establishment of disease models with genetic fidelity [34] and the simulation of drug responses that closely mimic in vivo outcomes [35]. Transcriptomic analyses have demonstrated that organoids exhibit a higher degree of similarity to native tissues [36]. Moreover, the development of various fundamental tissue organoids, such as vascular organoids, has made substantial contributions to bridging the gap between organoids of specific sites (e.g., brain organoids, liver organoids, and ovarian organoids) and physiological organs [37].

All in all, the rapid advancement of organoids has remedied the shortcomings of the models employed in traditional FRT studies. This review intends to succinctly and precisely summarize the fundamental paradigms of organoid establishment and their applications in female reproductive research. Furthermore, we summarize the cell sources during organoid establishment, the extracellular scaffolds utilized, and the requisite nutrient supplies. Additionally, we delineate the research progress of organoids that simulate the structures and functions of the ovary, the fallopian tube, the uterus, and the cervix, as well as the blastocyst and placenta organoids employed in recreating the embryo implantation process. We concurrently cover the utilization of organoids in the research of key diseases in the reproductive tract. It should be noted that although this review covers both benign and malignant endometrial conditions, its primary focus is on benign pathologies (e.g., Asherman’s syndrome, endometriosis, and adenomyosis), with a more concise discussion of cancers (endometrial and ovarian) in later sections. We hope to offer a refined summary and systematic organization for organoids, better facilitating their development and application in the domain of reproductive biology.

## 2. The Fundamental Constituents of Organoids

### 2.1. Cell Sources of Organoids

The cellular materials that compose organoids can be sourced from multiple cell types, thereby providing the most essential biological components for the construction of physiological and pathological disease models [38]. According to the source of the cells and the methods through which different cells give rise to organoids, these cells can be broadly categorized into three types: pluripotent stem cells (PSCs), primary cells, and cell lines.

PSCs have strong proliferation and differentiation abilities, which lay the foundation for the simulation of tissue genesis by organoids [39]. By supplying specific growth factors to organoids, the direction of their growth and differentiation can be artificially controlled, and this has been extensively utilized in the establishment of intestinal organoids [40]. In current research, the generation of organoids using PSCs mainly involves two implementation paths, with the primary difference lying in the number of PSCs transplanted. Firstly, PSCs are cultured in a monolayer. Under suitable culture conditions, these PSCs will spontaneously form aggregates known as embryoid bodies (EBs), which are then transferred to extracellular scaffolds to form organoids [41,42]. If organoids are generated from a single PSC, the formation of embryoid bodies (EBs) can be precluded by adding a rho-associated kinase pathway inhibitor (ROCK) to the culture medium prior to embedding the single PSC in an extracellular scaffold [43].

The organoids generated by using primary cells isolated from tissues exhibit certain biological features of mature tissues in vivo, such as autocrine, paracrine, or endocrine effects. The target tissues are cut into small fragments (with lengths of 1 mm and 2 mm), and various primary cells are obtained under the digestion of biological enzymes (trypsin or collagenase) [44,45,46,47,48,49]. Purified tissue-resident adult stem cells (ASCs), progenitor cells, or differentiated somatic cells can be obtained through operations like fluorescence-activated cell sorting (FACS), magnetic-activated cell sorting (MACS), and cell filters [50]. The isolated primary cells are subjected to in vitro culture within the extracellular scaffold, which supports their self-organization and development for the formation of organoids [38].

Organoids can also originate from cell lines. Cells of the cell lines are typically subjected to monolayer culture first, and, after reaching a certain density, they are inoculated into extracellular scaffolds for culturing to produce organoids [51]. Commercially available cell lines that can form organoids include BT-474 for breast cancer organoids [45], LIM1863 for colon cancer organoids [52], and BTS5 and BTS11 for trophoblast organoids [53]. The utilization of cell lines enables organoids to proliferate stably over an extended period and exhibit stable cellular characteristics of somatic cells. At present, organoids can also be formed from cells that have undergone gene editing. Through the application of the CRISPR-Cas9 technique, single guide RNAs (gRNAs) targeting specific genes can be engineered [54]. Through electroporation or transfection to introduce it into cells, transgenic cells with specific protein expression levels can be acquired [55,56]. Ultimately, the transgenic cells were cultivated together with the extracellular scaffold to obtain transgenic organoids. In contrast to the monolayer culture and utilization of transgenic cells, transgenic organoids can better simulate the effects of mutant cells on the surrounding cells and tissues, such as the changes in the spatial structure of cells that are typically difficult to observe in 2D culture.

The choice of which cells are employed to construct organoids constitutes a crucial factor influencing the outcomes, and each approach has its own merits and limitations. PSCs and cell lines can presently be obtained through commercial purchase for subsequent use, yet they are incapable of mimicking the characteristics of native tissues as effectively as primary cells. The principal drawbacks of obtaining organoids from primary tissues encompass the invasiveness of tissue biopsy and the complexity of cell isolation. Furthermore, as the majority of primary cells have a high degree of differentiation, the number of passages of the organoids established therefrom is significantly lower than that of PSCs and cell lines. Regarding organoids derived from transgenic cells, similarly to any genetically modified cell lines, a substantial loss of intracellular substances in the cells constituting the organoids occurs [57,58,59], rendering it arduous to sustain long-term culture and resulting in low efficiency [60].

### 2.2. The Growth Circumstances of Organoids

The extracellular scaffolds offer the basic structural support for the growth and proliferation of organoids [61]. In studies involving organoids, hydrogels are the predominant type utilized for 3D organoid culture (Table 1). Hydrogels are cross-linked hydrophilic polymer networks with a high water content and physicochemical properties similar to those of animal tissues, which enables hydrogels to ensure the exchange of nutrients and oxygen within the organoids [62,63]. Simultaneously, in light of the chemical structural characteristics of hydrogels, researchers can modify the physical properties or chemical composition of hydrogels in accordance with the distinct culture requirements of different organoids [64].

At present, the commonly employed hydrogels can be approximately classified into two major types: natural hydrogels and synthetic hydrogels. Recently, defined animal-free matrices have gained increasing attention for clinical applications due to their well-controlled composition and reduced batch variability. These include polyethylene glycol (PEG)-based hydrogels and modular click chemistry hydrogels, which allow for precise tuning of mechanical properties and biochemical cues while eliminating xenogenic components [65,66,67,68,69].

Natural hydrogels typically comprise diverse sugars, proteins, and other biological constituents, and they are extensively utilized in tissue engineering [62,70]. However, their clinical translation is often limited by animal-derived components and batch to batch variations. Conversely, the physical and chemical characteristics of synthetic hydrogels are stringently controlled artificially, fulfilling the requirements of diverse customization [71]. Among synthetic hydrogels, PEG-based systems are particularly promising for clinical use due to their biocompatibility, tunable properties, and regulatory approval history [72,73]. Click chemistry hydrogels offer additional advantages of modular assembly and spatiotemporal control over gelation [74,75].

Natural hydrogels can be categorized as protein hydrogels, polysaccharide hydrogels, and decellularized extracellular matrix hydrogels. Among these, Matrigel, derived from Engelbreth–Holm–Swarm (EHS) mouse sarcoma, is currently the biomaterial with the broadest application range, and it is considered the gold standard for organoid culture. Nevertheless, its animal origin and undefined composition pose challenges for clinical translation. Synthetic hydrogels encompass those based on poly lactic-co-glycolic acid (PLGA), polyethylene glycol (PEG), polycaprolactone (PCL), and RADA 16. They are materials featuring distinct compositions, cross-linking structures, degradation rates, and rheological properties [71]. Recent advances in bioorthogonal click chemistry have enabled the development of modular synthetic matrices that can be dynamically modified to mimic native tissue remodeling.

In the process of establishing a particular type of organoid, an appropriate extracellular scaffold should be furnished in accordance with the growth environment of its original cells so as to increase the growth rate and stability of the organoid. For clinical applications, special consideration should be given to animal-free, chemically defined systems that meet regulatory requirements while maintaining organoid functionality.

**Table 1:** Common types of hydrogels employed in organoid modeling.

Type	Classification	Main Ingredients	Application	Document
Natural hydrogel	Protein hydrogel	Matrigel	Intestinal organoids, liver organoids, pancreatic organoids, ovarian organoids, prostate organoids, endometrial organoids	[76,77,78,79,80,81,82,83]
		Collagen matrix	Intestinal organoids, liver organoids, lung organoids, brain organoids	[84,85,86,87]
		Fibrin matrix	Kidney organoids	[26]
		Silk fibroin matrix	Brain organoids, intestinal organoids	[88,89]
	Polysaccharide hydrogel	Alginic-acid-based matrix	Neural organoids	[90]
		Chitosan matrix	Intestinal organoids	[91,92]
	Decellularized extracellular matrix hydrogel	-	Kidney organoids, ovarian organoids, intestinal organoids, spinal cord organoids, and mammary gland organoids	[93,94,95,96,97,98]
Synthetic hydrogel	Poly lactic-co-glycolic acid hydrogel	Poly lactic-co-glycolic acid	Intestinal organoids, hepatic organoids	[99,100,101,102]
	Poly-caprolactone hydrogel	Polycaprolactone	Neural organoids	[103]
	Polyethylene glycol hydrogel	Polyethylene glycol	Intestinal organoids	[104]
	PuraMatrix	RADA 16	Brain organoids	[105,106]

### 2.3. Nutrient Supply for Organoids

Apart from extracellular scaffolds, organoids also demand diverse nutrients and signaling molecules during the culturing process, which are of vital significance for the stable expansion of organoids. Virtually all organoids from different cell sources have their distinctive nutritional requirements. Hence, whether the composition of their culture medium is correctly selected becomes a key precondition for the successful establishment of organoids. Furthermore, the various cell types and developmental stages encompassed in organoids during their development also have dissimilar culture requirements. The organoid culture medium consists of four main components, including the basal medium commonly used in monolayer cell culture, serum, antibiotics, and soluble factors. Soluble factors can be regarded as the most crucial component in the organoid culture medium, and it can be said that they determine the ultimate fate of organoids. Once the soluble molecules in the organoid culture medium bind to cell receptors, they activate or inhibit intracellular signal transduction processes to initiate cell differentiation or proliferation. In Table 2, we summarize the soluble factors that are frequently added to existing organoid culture media. Among these soluble factors, growth factors are generally expensive and unstable, while small molecule drugs may affect off-target pathways, resulting in poor reproducibility. Therefore, a study conducted experiments by combining biologics and small molecule drugs in organoid cultures and achieved positive results [107]. This offers novel perspectives for enhancing the culture conditions of organoids and reducing the expenditure of experiments.

## 3. The Application of Organoids in the Study of Reproductive Tract Physiological Functions

### 3.1. Ovary

**Figure 2:**
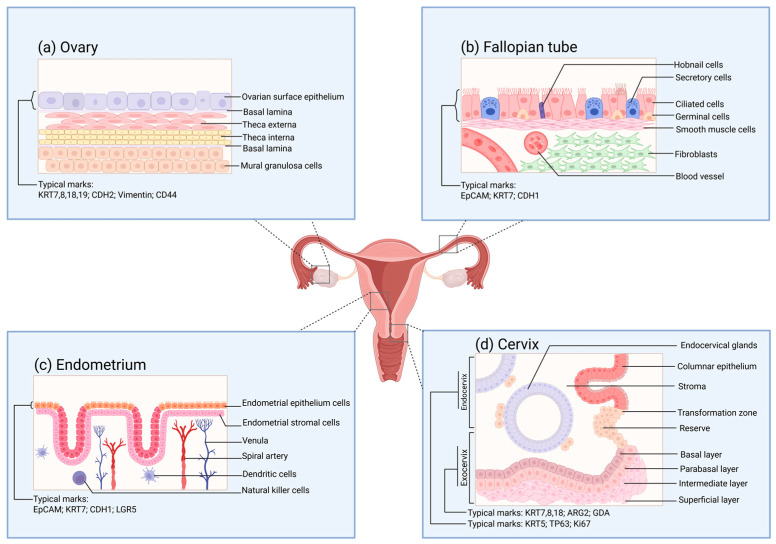
The surface structure of the functional layers of various parts of the female reproductive tract under normal conditions. (**a**) Schematic diagram of the tissue structure of the functional layer on the surface of the ovary. The cell markers of the ovarian surface epithelium are also provided in the figure. These markers are of great reference significance for verifying whether the organoids are constructed successfully. (**b**) Schematic diagram of the tissue structure of the functional layer on the inner side of the fallopian tube. The cell markers of the inner mucosal epithelium of the fallopian tube are also provided in the figure. (**c**) Schematic diagram of the tissue structure of the functional layer on the surface of the endometrium. The cell markers of the endometrial epithelium are also provided in the figure. (**d**) Schematic diagram of the tissue structure of the functional layer on the surface of the cervix. The cell markers of the endocervix and the exocervix are also provided in the figure.

The human ovary can be histologically classified into two parts: the ovarian parenchyma and the ovarian stroma [130]. The ovarian parenchyma constitutes the functional units of the ovary, namely, the ovarian follicles. The ovarian stroma is composed of supporting tissues, including nutrients and immune, nerve, and certain specific components [131,132]. The development, maturation, and release of oocytes are inseparable from the normal functioning of the ovary. Meanwhile, the ovary becomes one of the significant endocrine glands by secreting estrogen and progesterone to regulate the vital activities of the organism [133]. The quantity of oocytes that different female animals can generate throughout their lives, or the total number of follicles, is relatively fixed [134]. Ovulation takes place under the multiple regulations of the hypothalamic–pituitary–ovarian hormone axis (HPO) and paracrine cells, enabling the release of primary oocytes from the ovarian surface epithelium (OSE) (Figure 2a) [133]. The distinctive molecular characteristics of OSE allow it to self-repair after ovulation [135]. In this regard, the structural importance of the surface cells of the ovary and their plastic capabilities have aroused widespread research interest.

**Figure 3:**
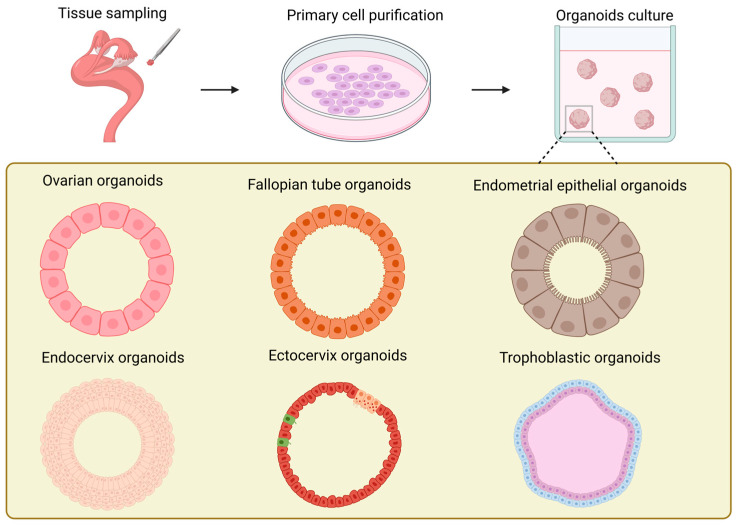
Schematic illustration of the generation and models of organoids in various female reproductive tracts.

In contrast to the ovarian stroma, studies on ovarian organoids (Figure 3) have been more concentrated on the ovarian surface epithelium [136]. The exploration of the construction conditions of ovarian organoids can be traced back to the study on the repair of ovarian surface wounds by P A Kruk et al. in 1992 [137]. Their research employed the established ovarian surface epithelial organoids to simulate the wound repair process after ovulation in the ovary. At the same time, it was discovered that the formation rate of the organoids was correlated with the quantity of human ovarian surface epithelial cells inoculated in the initial stage of each organoid and the number of fibroblasts in the collagen gel. Furthermore, the study proved that the formation of ovarian surface epithelial organoids was not influenced by epidermal growth factor, hydrocortisone, or the ratio of serum in the culture medium. It offered novel ideas and approaches for the study of other ovary-related diseases, such as cysts. In the research conducted by Kwong et al., the established in vitro organoids of normal human ovarian surface epithelial cells were analogous to the epithelial inclusion cysts in the human ovarian cortex, which are the origin cells of ovarian epithelial tumors. They utilized this model to investigate the relationship between chronic inflammation and the incidence rate of ovarian cancer [138]. However, its incapability of long-term culture constitutes its most significant application limitation. Ovarian organoids can also originate from female germline stem cells [139,140]. In contrast to the previously mentioned organoid models derived from primary cells, reproductive stem cell organoids possess endocrine functions and are capable of generating oocytes in vitro. Nevertheless, the maturation rate of oocytes produced from organoids remains rather low and awaits further improvement and enhancement.

The degree of physiological resemblance of ovarian organoids is continuously being refined, and exploration has begun of the pathological processes of ovarian diseases. All in all, for the investigation of physiological mechanisms, most ovarian organoids are typically established by first isolating ovarian surface epithelial cells from the ovaries of healthy animals or by achieving directed guidance and differentiation of reproductive stem cells in monolayer culture in vitro and then inoculating these two types of cells into extracellular scaffolds [141,142]. This method of constructing organoids that simulate the normal physiological state lays a cornerstone for the establishment of disease organoids. On this basis, the addition of relevant growth factors, such as inflammatory cytokines and tumor necrosis factor-α, enables healthy ovarian organoids to progress towards pathological states during the culture process, achieving the aim of simulating diseases [138]. This approach enables precise control over the influence scope of additives, facilitating research on disease-related regulatory pathways. Additionally, pathological organoids of the ovary can also be constructed by isolating primary cells from animals in a diseased state or cells from genetically engineered animals [143]. In contrast to the former, the difficulty of directly constructing organoids from cells in a pathological state will increase substantially, but the degree of conformity to the disease will also be enhanced significantly.

At present, the development of ovarian organoids is gradually advancing towards a more microscopic dimension. For example, studies have explored how growth factors facilitate the proliferation of stem cells in organoids [144]. Building on this, transcriptomic sequencing techniques have been increasingly incorporated into relevant research. This has enabled the revelation of the physiological mechanisms underlying cell proliferation within ovarian organoids [145]. These novel research findings will undoubtedly further drive the rapid progress of ovarian organoid research.

### 3.2. Fallopian Tube

For traditional natural fertilization, the transmission of reproductive gametes, fertilization, and the early development of embryos are all dependent on the role of the fallopian tubes [146]. The mucosa of the human fallopian tube (Figure 2b) is arranged in longitudinal folds and lined with a monolayer of the columnar epithelial structure. The main constituents of this structure are ciliated cells and secretory cells, while also including a small number of wedge-shaped cells and migratory cells [147]. Under the regulation of estrogen and progesterone, the fallopian tubes undergo periodic variations [148]. The expression levels of estradiol (E2) and progesterone receptors in the fallopian tube epithelium differ in accordance with the distinct stages of the ovarian cycle [149]. Meanwhile, such a change will also influence the epithelial structure of the fallopian tubes and the expression of cilia [150]. When the oocyte is released, the cilia will capture the oocyte and guide it to the junction of the ampulla and the isthmus for fertilization by sperm. Subsequently, the cilia will further direct the fertilized egg into the uterus for the implantation process. During this process, the secretions of the secretory cells will enhance the motility of the gametes and the fertilized egg [151].

In 2012, Paik et al. made the inaugural attempt to undertake three-dimensional culturing of fallopian tube epithelial cells (FTE). They embedded FTE cells in an extracellular matrix mixture of Matrigel and growth medium and successfully produced iterative spherical structures, which can be regarded as precursors of organoids [152]. The sign of the successful establishment of fallopian tube organoids (Figure 3) is generally considered to be the research of Kessler et al. [153]. The most prominent characteristic of this fallopian tube organoid lies in the completeness of its cellular composition. Owing to the existence of bipotent stem cells, the organoid can possess both secretory cells and ciliated cells through long-term proliferation and differentiation, and it can stably expand for over a year without obvious phenotypic alterations. This completeness enables the established fallopian tube organoid to have hormone responsiveness, significantly increasing its similarity to in vivo tissues. Thereafter, Ross et al. found that the volume of organoids derived from primary cells in the distal portion of the fallopian tube was significantly larger than that of organoids derived from cells in the proximal portion, which is in line with the morphology of cells in vivo [154]. They designated aldehyde dehydrogenase (ADLH) as a biomarker for the formation of FT organoids, thereby establishing a criterion for the subsequent identification of FT organoids.

The types of extracellular scaffolds for culturing FT organoids are constantly being renewed. FT organoids have been successfully cultivated in 3D thermoreversible gel polymer (TGP), and the organoids thus cultivated also bear the biological markers of FT [155]. It is notable that Chang et al. constructed an in vitro co-culture model encompassing FT organoids, mesenchymal FT stem cells, and umbilical cord endothelial cells. This assemblage, integrating different types of cells, offers a novel research modality for investigating the regeneration of fallopian tube epithelium and cancer-like transformation [156]. Additionally, in summary, it can be observed that the successful cultivation of fallopian tube organoids is inseparable from the important growth factors that support the paracrine signaling pathways, particularly Wnt and Notch. This is not only a crucial factor for the successful cultivation of fallopian tube organoids; it also offers significant reference significance for the establishment of other types of organoids and organoid co-culture systems.

### 3.3. Uterus (Endometrium)

The endometrium, as a crucial structure participating in the pregnancy process, shoulders the functions of nurturing the fetus and generating menstruation. The endometrium (Figure 2c) is distributed on the inner surface of the uterine cavity. Abnormalities in its physiological functions may give rise to disorders like pregnancy disorders and implantation failure [157,158]. The human endometrium consists of two components, namely, the epithelial layer and the basal layer. Among them, the epithelial layer can be further subdivided into glandular epithelium and luminal epithelium. The nourishment of the endometrium mainly originates from the blood transported within the blood vessels. The blood vessels distributed in the endometrium are constituted by endothelial cells covered by the basement membrane [159]. The luminal epithelium of the endometrium offers the implantation site for embryo attachment. In contrast to the spatial structural significance of the luminal epithelium of the endometrium, the glandular epithelium assumes more physiological functions. The glandular cells encompassed by the glandular epithelium consist of cells situated near the myometrium (the so-called basal layer cells) and functional cells in the upper layer. During the early stages of pregnancy, the secretions of the glandular cells exert a crucial role in the interaction between the mother and the fetus and in supporting embryo development [160].

The establishment of endometrial organoids (Figure 3) can originate from primary cells of the endometrium at various periods, such as the proliferative phase, the secretory phase, the gestational period, etc. [82]. Endometrial organoids can be categorized into endometrial epithelial organoids and endometrial epithelial–stromal organoids in accordance with their cellular composition [76,82,161,162]. The three-dimensional culture of endometrial epithelium originated in 1988 [163]. Rinehart et al. initially isolated primary epithelial cells of the endometrium and subsequently transferred them into Matrigel, successfully obtaining spherical structures with epithelial cell morphology. In 2017, two groups of researchers established endometrial epithelial organoids from the endometrium of humans and mice obtained via biopsy [76,82]. All of these organoids originated from the endometrial epithelium and precisely mimicked the histological characteristics of the glandular epithelium in vivo. They formed a cavity with cell polarity and possessed the same characteristics as the endometrial glands in vivo, such as the expression of markers (MUC 1, ECAD, KRT 7, EPCAM, FOXA 2, Pan-KRT) and hormone receptors (E2, P4), as well as similar structures (cilia) [32]. By regulating the degree of expression of the markers SPP1, PAEP, LIF, and 17HSDβ2 in organoids [82], endometrial organoids can be guided to differentiate into secretory phase organoids and pregnancy phase organoids, respectively, to simulate the endometrium in the proliferative phase and the pregnancy phase. In 2019, Haider et al. found that the ciliated cell phenotype encompassed by endometrial organoids could be modulated through the application of hormones and NOTCH signaling [164]. Furthermore, in comparison with epithelial organoids, epithelial–mesenchymal organoids intermingled with stromal cells are more histologically analogous to the endometrium [161,162]. At present, the most up-to-date endometrial organoids have begun to endeavor to incorporate vascular endothelial cells into the construction system, further approaching perfection for endometrial organoids [165].

During the culture of endometrial organoids, apart from the widespread utilization of Matrigel, researchers are likewise actively attempting to employ other biomaterials. Francés-Herrero et al. reported that the hydrogel of decellularized porcine endometrium is capable of enhancing the cell proliferation and stability of organoids and preserving the characteristics of stem cells [166]. Endometrial organoids have also been successfully cultured in hydrogels derived from the endometrium of cattle and humans [167]. The cell sources of endometrial organoids are also trending towards diversification. For example, menstrual blood is becoming a non-invasive source during the process of organoid establishment [168]. Similarly to tissue biopsies, these organoids originated from menstrual blood possess similar biological features, such as cell proliferation, cell phenotype, and gene expression. In contrast to organoids established from other parts of the female reproductive tract (FRT), endometrial epithelial organoids can also be directly established from tissue blocks [76,82]. After being digested by collagenase, the endometrial tissue releases glandular segments. The obtained glandular-like structures can be embedded in the Matrigel and undergo long-term culture by adding a specific organoid culture medium.

Currently, the co-culture model of endometrial epithelial–stromal organoids bears an extremely high resemblance to the endometrium in the human body, and researchers have commenced attempts to add blastocyst-like entities to simulate the process of embryo implantation [169]. However, currently, the co-culture models of endometrial organoids and other organoids are still a long way from achieving the aim of in vitro reproduction [161,170]. But, this does not diminish the potent role and substantial value of organoids as a tool for studying the physiology of the endometrium.

### 3.4. Cervix

The cervix is a cylindrical structure that connects the uterus and the vagina, and the cervical canal within it serves as a passage between the two sides [171]. The cervix can be classified into the endocervix and the ectocervix based on its spatial position (Figure 2d), among which the former is in direct contact with the vagina. At the cellular level, the epithelial cells and stromal cells separated by the basement membrane are the major cells constituting the cervix. The morphology of the epithelial cells varies according to the distribution area. The endocervix is covered by columnar epithelial cells; meanwhile, the ectocervix is covered by continuous stratified non-keratinized squamous epithelial cells, encompassing four layers: the superficial layer, the intermediate layer, the parabasal layer, and the basal layer. Squamous epithelial cells in different layers can express specific keratins [76]. The glands situated within the cervix secrete a kind of mucus, which is called cervical mucus [172]. The properties and quantity of this mucus, similarly to those of the endometrium, are both affected by ovarian function and exhibit distinct cyclical variations.

The advent of cervical organoids (Figure 3) occurred relatively later than that of other parts of the reproductive tract [173]. Cervical organoids can be categorized into two kinds: endocervical organoids and exocervical organoids. Both can be derived from human cervical tissues and undergo long-term culturing [174,175,176]. The cervical organoids established in these studies excellently mimic the phenotypic characteristics of the original tissues: In terms of gene expression, the distinctive mucin expression of endocervical organoids and the keratin expression of exocervical organoids are greatly in accordance with the in vivo tissues. Structurally, not only cystic tissues of endocervical cells are generated, but also the stratification of exocervical cells that can be maintained across generations is reproduced [176]. Currently, the role of WNT signal transduction in the establishment of cervical organoids remains contentious. Lõhmussaar et al. assert that in the presence of the WNT signaling pathway enhancer RSPO1, ectocervical organoids can be cultivated [176]. Conversely, Chumduri et al. discovered that the absence of WNT3a and the presence of RSPO1 are indispensable for the growth and passage of ectocervical organoids [175]. All in all, the rapidly evolving cervical organoids offer potent tools for studies of HPV and tumor mechanisms, drug research and development, as well as personalized medicine.

### 3.5. Placenta

The placenta lies between the uterine wall and the fetus, functioning as a bridge connecting the maternal and fetal circulations [177]. It originates from the fusion of the chorion and the decidua basalis subsequent to the implantation of the fertilized egg. As the pregnancy advances, it gradually develops and reaches maturity, with a complete structure formed at approximately the 12th week of gestation. A structurally intact placenta consists of the amnion, the villous chorion (also referred to as the plexiform chorion), and the decidua basalis. The decidua basalis is firmly connected to the uterine wall of the mother, offering a stable anchoring point for the placenta. The amnion covers the fetal aspect of the placenta, safeguarding the fetus from external injuries. The chorion, rich in blood vessels, is not only the primary site for material exchange but also exerts significant influences on the pregnancy process in aspects like promoting the synthesis of estrogen and progesterone, maintaining pregnancy, and facilitating the growth of the fetus–placenta unit. Trophoblast cells, being the major constituent cells of the placental chorion, assume the principal duties and can be categorized into internal villous cytotrophoblasts (VCT), external syncytiotrophoblasts (SCT), and extravillous trophoblasts (EVT) based on their distinct functions [178].

The establishment of trophoblast organoids from placentas in the early stages of pregnancy (Figure 3) possesses the capacity to simulate placental villi [179,180]. Meanwhile, if WNT signal transduction is strengthened during the growth of trophoblast organoids, they can form internal syncytial masses and external proliferative VCT. Meanwhile, in the case of no addition of WNT activators or when employing the differentiation medium for 2D culture of human trophoblast stem cells (hTSC) [181], trophoblast organoids are capable of generating EVT. They have exhibited remarkable genetic stability during the process of long-term culture, and their transcriptional levels and epigenetic characteristics are highly similar to those of trophoblast cells generated in the early stages of pregnancy. Additionally, through the study of the secretome of trophoblast organoids through mass spectrometry, numerous expression products of the in vivo placenta can be detected, and EVT demonstrates invasive behavior in Matrigel that is comparable to that in vivo [180]. Recently, the generation of placental villous organoids (PVOs) from healthy and pathological pregnancy states through air–liquid interface culture has emerged as a new tendency in the development of placental organoids. In contrast to trophoblast organoids, PVOs not only encompass cytotrophoblast cells that can directly self-renew and differentiate; they also incorporate immune cells, offering novel research perspectives for the investigation of placental barrier and immune functions [182]. By constructing placental organoids in vitro, researchers can simulate the early development process of the placenta and study its key mechanisms, such as different cell differentiation, material exchange, and immune functions. For example, in recent research, components related to the immune system have been further added to the organoids that mimic the placenta [182,183,184,185]. This significantly increases the level of mimicry of the placental structure in vitro. All in all, these are of great significance for improving the success rate of pregnancy.

## 4. The Application of Organoids in the Study of Pathological Mechanisms of the Reproductive Tract

### 4.1. Asherman Syndrome

**Figure 4:**
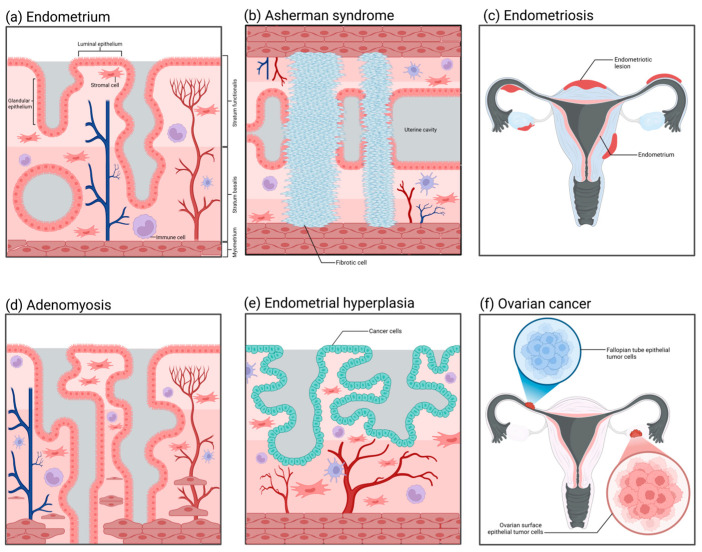
The histopathological structures of common diseases in the female reproductive tract. (**a**) Normal endometrium: It is divided into the basal layer and the functional layer, containing epithelial cells, stromal cells, blood vessels, and immune cells. (**b**) Intrauterine adhesions (Asherman syndrome): Adhesions form within the uterine cavity, with the functional layer epithelium replaced by fibrous tissue, lacking blood vessels and glands. (**c**) Endometriosis: Endometrial-like tissue grows outside of the uterus. (**d**) Adenomyosis: Endometrial glands and stroma invade the myometrium, causing the uterus to enlarge. (**e**) Endometrial hyperplasia: Abnormal proliferation of endometrial epithelial cells, which may develop into endometrial cancer. (**f**) Ovarian cancer: Abnormal proliferation of ovarian cells forms a tumor, which can spread to other areas.

Asherman syndrome (Figure 4b), also referred to as intrauterine adhesions, has an incidence rate affected by multiple factors, presenting diverse clinical symptoms and distinct pathological characteristics. The incidence of Asherman syndrome is approximately 1.5%. Nevertheless, repeated curettage after miscarriage can markedly increase its occurrence rate, sometimes up to 39%. The incidence of intrauterine adhesions resulting from multiple uterine cavity operations can also reach as high as 25% to 30%, and the recurrence rate can be as high as 66% [186,187]. Owing to the decreased volume of the uterine cavity, the fertilized ovum may be unable to implant and grow, and therefore the patient may also present with infertility and recurrent miscarriages [188]. The principal etiology of Asherman syndrome is uterine cavity surgeries, such as artificial abortion, curettage, removal of intrauterine contraceptive devices, etc. These procedures may injure the endometrium, resulting in the formation of intrauterine scars and chronic endometritis and subsequently inducing adhesions. Furthermore, factors like infection and decreased estrogen levels may also contribute to the occurrence of this disorder [189,190,191,192]. Currently, although organoid technology has already been utilized in research on Asherman syndrome, it is more frequently employed as an auxiliary approach for regeneration [193,194]. How to enable the endometrium to regenerate and restore its normal thickness is currently a research focus of Asherman syndrome. Based on this, researchers have induced endometrial injury through artificial mechanical damage or chemical toxicity and established a preliminary organoid model in rodents [195,196,197,198]. In contrast to the imperfect 3D organoid models, healing models that can precisely determine the promoting effect of compounds on wounds are utilized more prevalently [199,200]. Such models can simulate scarring, fibrosis, and even adhesion processes on the endometrial surface. The continuous progression of technology has made it feasible to establish standardized organoid models of Asherman syndrome.

### 4.2. Endometriosis

The incidence of endometriosis (Figure 4c) is relatively low among healthy women at approximately 10% to 15%. Nevertheless, among patients with chronic pelvic pain or dysmenorrhea, the incidence rises significantly, potentially reaching 20% to 90%. Especially among women of reproductive age, the incidence of this disorder is relatively high, with approximately 25% to 50% of women possibly afflicted by it [201,202]. Regarding the etiology of endometriosis, Sampson postulated that during menstruation, certain endometrial debris might reflux through the fallopian tubes into the peritoneal cavity and implant and grow in locations like the ovaries and the pelvic peritoneum, thereby initiating endometriosis. Nevertheless, although the majority of women have retrograde menstruation, only a minority develop endometriosis, suggesting that other factors also contribute to the pathogenesis [203]. Furthermore, ectopic endometrial tissue demonstrates abnormal responses to estrogen and progesterone. Such abnormal hormonal responsiveness might also be one of the crucial factors leading to the occurrence and progression of endometriosis [204,205,206,207]. The mature application of organoid technology in the pathological study of endometriosis can be traced back to research conducted in 2019 [208]. The study sampled from ectopic endometrium, corresponding eutopic endometrium, and healthy endometrium to establish three distinct types of organoids. The ectopic organoids possessed a thicker epithelial layer compared to the other two, and, when transplanted into mice, they induced the generation of endometriosis-like lesions. Simultaneously, it was discovered that the ectopic organoids exhibited obvious differences in gene expression, such as ECM-receptor interaction genes, adhesion and invasion genes, as well as PI3K-AKT pathway genes, among others. On the basis of that study, subsequent research has successively verified that endometriosis organoids can precisely recapitulate the proliferative pathological alterations of patients’ endometrium and the characteristics of eutopic and healthy endometrium [209,210,211]. Furthermore, the establishment of endometriosis stromal cell spheroids has successfully recapitulated the invasion process of this disease in vitro [212]. The utilization of organoid and spheroid techniques has expedited research progress on the pathological mechanism of endometriosis significantly.

### 4.3. Adenomyosis

Adenomyosis (Figure 4d) is a common gynecological disorder that is recalcitrant to cure. It has been reported in studies that approximately 82% of patients with adenomyosis in the United States currently opt for hysterectomy, and 37.6% of patients chronically use painkillers to alleviate pain [213]. The pathological characteristic of adenomyosis under the microscope is the occurrence of endometrial glands and stroma in the uterine myometrium. These glands and stroma grow within the myometrium, forming diffuse or localized lesions. This kind of lesion is often more extensive and evident in the posterior uterine wall, resulting in thickening of the posterior uterine wall. The glands and stroma that are ectopic in the uterine myometrium are also influenced by the menstrual cycle, presenting as abnormal bleeding and dysmenorrhea clinically. Moreover, if adenomyomas are present, there will be significant hyperplasia of smooth muscle around the glands, and tumor nodules in the myometrium can be observed [214,215]. The three-dimensional disease model of adenomyosis established by Mehasseb et al. in 2010 could be regarded as the antecedent of adenomyosis organoids [216]. The uterine myocytes of this model were cultivated within Matrigel; meanwhile, the endometrial stromal cells were inoculated on the top layer of the Matrigel. The aim of this structure was to simulate the pathological process of stromal cells invading myocytes. The invasiveness of stromal cells in the co-culture system was markedly higher than that of the control group, and the difference was statistically significant. In subsequent studies, by using this model, Taylor et al. discovered that estradiol and estrogen receptor modulators could enhance the invasiveness of endometrial stromal cells [217]. This adenomyosis model not only offers an excellent idea and research basis for the future development of organoids in this disorder; it also reveals its considerable potential in cell invasion assays, hormone effects, and drug screening.

### 4.4. Endometrial Hyperplasia and Endometrial Cancer

Nowadays, the number of new cases of endometrial cancer (Figure 4e) is predicted to surpass 500,000 per annum [218,219]. Generally, endometrial cancer is categorized into two types, namely, type I endometrial cancer and type II endometrial cancer. The pathological type of type I endometrial cancer (hormone-dependent endometrial cancer) is mostly endometrioid adenocarcinoma, and the patients usually are younger and have a better prognosis. The etiology of type I endometrial cancer is mostly associated with estrogen [220,221]. When there is excessive secretion of estrogen without the protection of progesterone, it will exert excessive stimulation of the endometrium, resulting in a prolonged state of excessive hyperplasia of the endometrium, which may further progress to endometrial cancer. Patients may concurrently present with risk factors, such as obesity, hypertension, menstrual irregularities, and abnormal ovulation. The pathological morphology of type II endometrial cancer (non-hormone-dependent endometrial cancer) belongs to a rare type. Patients with this type of disease are mostly elderly women, with high malignancy of the tumor and a poor prognosis. Its pathogenesis has no definite relationship with estrogen and is mostly associated with gene mutations [222]. The application of organoid technology in the study of endometrial tumors has been extremely prevalent. The marker of the successful establishment of endometrial cancer organoids can be traced back to research conducted in 2017 [223]. The endometrial cancer organoids established in that research reproduced such pathological characteristics as disordered epithelial structures and disrupted basement membrane states that in vivo tumors would present. Subsequently, the organoids originated from endometrial tumors established by Boretto et al. could grow and be passaged stably over a long period, and their genomes also exhibited long-term stability and replicated the histological and morphological features of endometrial tumors [208]. Studies conducted thus far have indicated that organoids derived from the primary tumor tissues of the endometrium can precisely simulate the significant genetic, histological, and expression characteristics exhibited by tumors in vivo [81,82,224,225]. Meanwhile, the organoids established corresponding to endometrial hyperplasia and Lynch syndrome are capable of maintaining the mutations that occur in the tissues in vivo [208].

### 4.5. Ovarian Cancer

At present, in addition to establishing mature organoid models in the research field of endometrial cancer, researchers have also utilized organoid technology in the exploration of the pathological mechanisms of cancer diseases in other parts of the reproductive tract. Ovarian cancer (Figure 4f), as a non-single-type heterogeneous disease, comprises multiple tumor types with different biological characteristics, clinical manifestations, treatment responses, and prognostic outcomes. Among these types, high-grade serous ovarian cancer (HGSOC) is the most fatal and common one [226]. HGSOC is notable for its significant aggressiveness, rapid growth rate, and poor overall survival rate, which render it a key subject of concern in the research and treatment of ovarian cancer. The putative pathological origin of HGSOC mainly resides in the fallopian tube epithelium (FTE) and the ovarian surface epithelium (OSE). Research indicates that genetic mutations in fallopian tube epithelial cells, particularly the inactivation of the Tp53 and RB families, may result in the occurrence of HGSOC. These mutations may trigger abnormal proliferation and differentiation of cells, ultimately giving rise to tumors [227]. Another supposition is that HGSOC stems from the surface epithelial cells of the ovary [228]. Likewise, these cells might also experience genetic mutations, thereby resulting in the malignant transformation of the cells. The surface epithelial cells of the ovary are distinct from the epithelial cells of the fallopian tube in terms of morphology and biological characteristics; however, both possess the potential for malignant transformation. The organoids derived from the surface epithelial cells of the ovary can simulate the progression and metastasis of HGSOC in in vitro experiments, lending support to this origin hypothesis [229]. Nevertheless, such organoids are deficient in their capacity for long-term expansion.

In a study in 2019, Kopper et al. successfully established disease models of ovarian organoids with precancerous abnormal lesions and malignant ovarian cancer organoids that could be cultured for a long time [230]. These organoids exhibit cellular morphological characteristics similar to those of the corresponding tumors in vivo, diverse cell phenotypes (PAX8+, TP53+), and differentially expressed genomic profiles (KRAS, BRAF, cell cycle genes, and TP53 mutations). The fallopian tube organoid model that supports the alternative hypothesis that HGSOC originates from the fallopian tube epithelium can be obtained through knockdown of TP53, PTEN, and RB [231]. Recent advances have further demonstrated their utility in drug screening platforms, capturing tumor heterogeneity, and serving as patient avatars for personalized therapy [232,233,234,235].

## 5. The Development Prospects of Reproductive Tract Organoids

**Figure 5:**
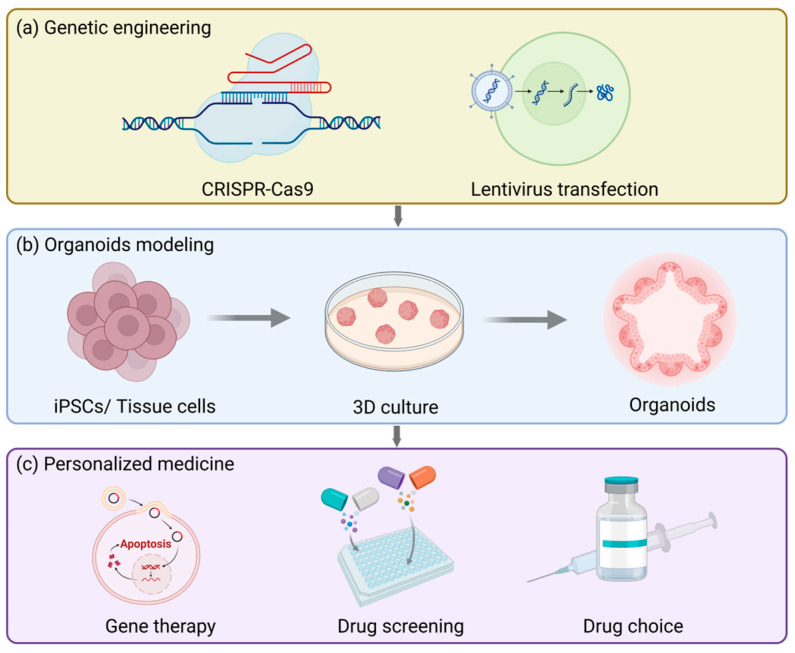
The future applications and prospects of reproductive tract organoids. (**a**) Organoid models can be used to study the physiology and pathology of the reproductive tract and also for genetic engineering, such as CRISPR/Cas9 gene editing or lentiviral transfection. (**b**) The basic procedure for establishing organoids. (**c**) Organoids can be used to test drug responses, accelerate drug development and screening, and assist patients with rare diseases in gene therapy.

Gene engineering techniques (Figure 5a), particularly the CRISPR-Cas9 system, can offer precise gene editing approaches for organoid modeling, allowing researchers to conduct operations like gene knockout, insertion, or site-directed mutagenesis in organoids. For example, in the previously employed process of establishing a fallopian tube organoid model targeting ovarian cancer, specific mutations were introduced to investigate the etiologies of endometrial and cervical cancers [230]. Furthermore, gene engineering techniques can also be integrated with lentiviral transduction and the PiggyBac platform to realize stable expression of transgenes. These platforms are not only applicable to standard/inducible overexpression and knockout but also capable of introducing specific gene mutations that are inadequately represented in patient populations, generating highly clinically relevant preclinical organoid models. This significantly broadens the promising prospects of personalized medicine and is particularly crucial for resolving the issue of female infertility.

Currently, the main factors contributing to female infertility mainly consist of two categories: pelvic factors and ovulation disorders. In vitro fertilization (IVF) is the main solution opted for by infertile patients. Nevertheless, even after undergoing the same hormonal treatment, the cause of the disease remains unknown in approximately 30% of cases [236]. The function of organoids in personalized medicine for infertility primarily resides in the fact that they can be established from endometrial biopsies of these patients, thereby investigating how individual variations influence different outcomes under the same treatment context and ultimately customizing treatment regimens for patients in accordance with the actual circumstances (Figure 5b). This concept can be applied in the treatment of endometriosis [237]. Furthermore, the ovarian epithelial organoid model is beneficial for the treatment of polycystic ovary syndrome, thereby mitigating the adverse effects of the commonly employed superovulation treatment methods nowadays [238].

It is notable that organoids originated from the pathological tissues of patients can be cryopreserved at low temperatures and subsequently thawed and resuscitated when necessary. This preservation pattern is conducive to the screening of preclinical drugs (Figure 5c) and can ultimately be utilized in personalized medicine [81,208,230,239,240]. When pathological model-derived organoids of ovarian cancer were treated with platinum/taxane, the sensitivity and drug resistance exhibited by the organoids were in accordance with the grading of the tumors in vivo [230]. Apart from directly simulating the drug effects in vitro, disease organoid models can also be transplanted into experimental animals, and then the drug responses can be evaluated [230]. Similar to this, the resistance of endometrial cancer organoids to cisplatin and paclitaxel reflects the clinical responses of patients to treatment, which is conducive for doctors to adjust treatment regimens and drug choices for patients [224]. All in all, because organoids originate from humans themselves, they can minimize the physiological differences among species and improve the accuracy of drug screening. Furthermore, organoids can be cultivated for a long time and support high-throughput drug screening, which is of vital importance for assessing drug efficacy and safety.

## 6. Conclusions

The female reproductive tract (FRT) is central to women’s health and reproduction, yet its regulatory mechanisms remain incompletely understood. While traditional in vitro and in vivo models face inherent limitations, organoid technology has emerged as a transformative tool enabling the reconstruction of FRT tissues (e.g., ovary, endometrium, fallopian tube, and cervix) with remarkable architectural and functional fidelity. These models recapitulate dynamic physiological processes and disease phenotypes, offering unprecedented insights into FRT physiology and pathology.

The contributions of organoids to female reproductive research can be encapsulated into several aspects. Foremost, and most significantly, they have facilitated the discovery of reproductive physiological mechanisms. Organoids have clarified hormone response signals (for example, endometrial regeneration [241]), cell interactions in endometriosis [242], and the mutational landscape in gynecological cancers. Regarding disease modeling, patient-derived organoids are capable of recapitulating pathological processes, including Asherman syndrome and the heterogeneity of endometrial cancer. This ability enables drug screening and the implementation of personalized treatment strategies. In addition, in the context of assisted reproduction, investigations into organoid-mediated support for embryo implantation [165] and trophoblast–endometrial crosstalk [243,244] have further propelled the advancement of infertility research. Nevertheless, to fully achieve clinical translation, it is imperative to tackle persistent challenges in the modeling and application of organoids. Existing models frequently lack vascularization and immune components, thereby constraining the investigation of microenvironmental interactions. Simultaneously, there is an absence of standardized protocols for organoid establishment and established metrics for assessment. These issues pose substantial barriers to the further expansion of the applicability of organoids.

To this end, we propose that future efforts in the development of organoids should prioritize integration with other technologies. For example, the integration of FRT organoids with microfluidics [245,246] or 3D bioprinting [247,248,249] can be explored to enhance physiological relevance. In the meantime, the reproductive research community ought to formulate standards for organoid modeling procedures. In addition, researchers can also promote the establishment of organoid biobanks and clinical trials for drug testing [208]. We are convinced that with the advancement of organoid systems, they will enhance our comprehension of FRT biology, expedite the development of targeted therapeutics, and, ultimately, close the gap between laboratory research and clinical applications.

## Figures and Tables

**Table 2:** Commonly added soluble factors in organoid culture media.

Substance	Function	Document
R-Spondin-1 (RSPO-1)	Wnt signaling pathway agonists	[108,109,110]
Nicotinamide	Inhibit cell differentiation	[54,110,111,112]
N-acetyl-L-Cysteine	ROS inhibitors, with antioxidant effects	[113]
Noggin	Regulate cell differentiation, proliferation, and apoptosis	[35,111,112,113,114]
Epithelial Growth Factor (EGF)	Promote cell proliferation and differentiation	[54,112,115]
Hepatocyte growth factor (HGF)	Promote the growth of gastric organoids and crypt organoids	[35,116,117,118]
Fibroblast growth factor (FGF)	Regulate cell proliferation and differentiation	[54,110,118,119,120,121]
Transforming growth factor alpha (TGFα)	Regulate stem cell differentiation	[42]
Gastrin	Participate in the proliferation and differentiation of gastric epithelial cells	[35,54,112]
Bone morphogenetic protein 4 (BMP 4)	Regulate the hormone levels of endocrine cells	[122,123,124]
Wnt	Promote the growth, proliferation, and differentiation of stem cells and inhibit apoptosis	[108,110,112,124]
A83-01	Promote cell proliferation, prevent cell differentiation, maintain stem cell pluripotency, and inhibit cell apoptosis and senescence	[110,120,125]
Y-27632	Inhibit embryonic stem cells and promote self-renewal and proliferation of stem cells	[54,110,126]
CHIR-99021	Activate the Wnt signaling pathway	[54,127]
SB 431542	Enhance the proliferation capacity of epithelial cells	[127,128]
L-ascorbic acid	Inhibit cell apoptosis	[129]
B27	Inhibit cell differentiation	[54,110,119,127]
HEPES	Maintain the osmotic pressure stability of the culture system and provide additional buffering capacity	[108,110,120,127]

## Data Availability

Not applicable.

## Abbreviations

The following abbreviations are used in this manuscript:

FRT	Female reproductive tract
BDNF	Brain-derived neurotrophic factor
IGFBP-1	Insulin-like growth factor binding protein-1
PSCs	Pluripotent stem cells
EBs	Embryoid bodies
ROCK	Rho-associated kinase pathway
ASCs	Adult stem cells
FACS	Fluorescence-activated cell sorting
MACS	Magnetic-activated cell sorting
gRNAs	Guide RNAs
EHS	Engelbreth–Holm–Swarm
PLGA	Poly lactic-co-glycolic acid
PEG	Polyethylene glycol
PCL	Polycaprolactone
RSPO-1	R-Spondin-1
EGF	Epithelial Growth Factor
HGF	Hepatocyte growth factor
FGF	Fibroblast growth factor
TGFα	Transforming growth factor alpha
BMP 4	Bone morphogenetic protein 4
HPO	Hypothalamic–pituitary–ovarian hormone axis
OSE	Ovarian surface epithelium
FTE	Fallopian tube epithelium
ADLH	Aldehyde dehydrogenase
TGP	Thermoreversible gel polymer
VCT	Villous cytotrophoblasts
SCT	Syncytiotrophoblasts
EVT	Extravillous trophoblasts
hTSC	Human trophoblast stem cells
PVOs	Placental villous organoids
HGSOC	High-grade serous ovarian cancer
IVF	In vitro fertilization
